# A Method for Using Cell-Penetrating Peptides for Loading Plasmid DNA into Secreted Extracellular Vesicles

**DOI:** 10.3390/biom13121751

**Published:** 2023-12-06

**Authors:** Jekaterina Nebogatova, Heleri Heike Härk, Anett Puskar, Ly Porosk, Paolo Guazzi, Moataz Dowaidar, Ülo Langel, Kaido Kurrikoff

**Affiliations:** 1 Institute of Technology, University of Tartu, Nooruse 1, 50411 Tartu, Estonia; 2 HansaBioMed Life Sciences Ltd., Mäealuse 2/1, 12618 Tallinn, Estonia; 3 Department of Bioengineering, King Fahd University of Petroleum and Minerals (KFUPM), Dhahran 31261, Saudi Arabia; 4 Interdisciplinary Research Center for Hydrogen and Energy Storage (IRC-HES), King Fahd University of Petroleum and Minerals (KFUPM), Dhahran 31261, Saudi Arabia; 5 Department Biochemistry and Biophysics, Stockholm University, S. Arrheniusv. 16B, Room C472, 106 91 Stockholm, Sweden

**Keywords:** extracellular vesicles, gene therapy, nucleic acid therapeutics, drug delivery system, cell-penetrating peptides

## Abstract

The low bioavailability and high toxicity of plasmid DNA (pDNA)-based therapeutics pose challenges for their in vivo application. Extracellular vesicles (EVs) have great potential to overcome these limitations, as they are biocompatible native cargo carriers. Various methods for loading pDNA into EVs, including electroporation, sonication, and co-incubation, have been previously investigated, but their success has been questionable. In this study, we report a unique method for loading EVs with pDNA through transient transfection using cell-penetrating peptides (CPPs). With this method, we found a 10^4^-fold increase in the expression levels of the luciferase reporter protein in recipient cells compared to the untreated cells. These data point to the high transfection efficacy and bioavailability of the delivered encapsulated nucleic acid. Furthermore, the in vivo experimental data indicate that the use of pDNA-loaded EVs as native delivery vehicles reduces the toxic effects associated with traditional nucleic acid (NA) delivery and treatment.

## 1. Introduction

Gene therapy is a promising treatment strategy for common conditions such as cancer, hemophilia, or cystic fibrosis [1,2,3,4]. This approach is based on the intracellular introduction and expression of genetic material, such as pDNA. However, pDNA is a relatively large molecule with a very high negative charge density. These properties, along with the limitations of traditional pDNA delivery methods previously employed for in vitro applications, present challenges for the wider gene therapy application in vivo.

Several approaches, such as viral vectors, liposomes, and cationic polymers, have been used to tackle issues like toxicity, low efficacy, clearance, and limited ability to overcome biological barriers [5,6,7,8,9]. Despite the progress in pre-clinical studies, there is a need for drug delivery systems with improved efficacy, biocompatibility, and safety for clinical applications [5,10]. The reasons behind the failure of these methods during their transition from in vitro to in vivo applications vary depending on the method. For example, electroporation is successfully used for intracellular pDNA delivery in vitro. However, it has limited in vivo application due to its hindered access to the target tissue, and it may cause local tissue inflammation [11,12]. 

The utilization of pDNA-loaded EVs has great potential to overcome the limitations associated with the clinical application of traditional pDNA delivery methods. EVs are small membranous entities around 50–150 nm in diameter that are excreted by all cell types. EVs mediate cell-to-cell interactions through the intercellular transport of RNA, DNA, proteins, and lipids [13]. Unlike traditional pDNA delivery vehicles, EVs are natively produced and are fully biocompatible. Furthermore, EVs have been found to efficiently cross biological barriers [14]. 

In order to fully exploit the potential of EVs, they need to be loaded with the desired cargo in a manner that does not compromise their delivery capability. EV-loading strategies can be categorized into pre-isolation and post-isolation loading. In this work, we focus on the development of a pre-isolation method by leveraging our expertise rather than the optimization and validation of other methods.

In the pre-isolation strategy, first, genetic material is introduced into the cells; this process is called transfection. During transfection, a portion of the cargo is naturally encapsulated into excreted EVs, which are subsequently isolated and purified from the conditioned media. Several methods of pre-isolation EV loading with mRNA are reported, such as cell-penetrating peptides, transfection reagents, and EXOtic system [13,15,16,17,18,19]. Previous attempts at loading pDNA into EVs through transient transfection have encountered challenges [20]. In the case of pre-isolation loading, the integrity of vesicles is preserved, as there is no need to damage the EV membrane during cargo loading. Considering this factor, along with our expertise in the transient transfection of cell culture, we chose the pre-isolation strategy as the foundation for our proposed method of loading a cargo into EVs. 

Conversely, in the case of the post-isolation strategy, EV loading is performed after the isolation and purification of the vesicles and includes methods such as electroporation, co-incubation with cargo, sonication, extrusion, and chemical transfection. For example, electroporation, used for pDNA-based therapeutic delivery, demonstrated minimal changes in gene expression and led to the upregulation of inflammatory molecules [21]. EVs used for post-isolation loading contain material from donor cells, and integrity is compromised during this process, making it a less preferable choice for further investigation.

In the context of the pre-isolation EV-loading strategy, first, cells have to be provided with pDNA to be encapsulated. Different chemical and physical methods were developed for the transient transfection of the cells. Our field of expertise lies in the application of CPPs for the delivery of NA in vivo and in vitro. CPPs are relatively short peptides that condense NA through non-covalent interactions and through this process form complexes. Despite the advantages of the utilization of CPPs for intracellular delivery, high toxicity and the lack of targeting ability make it an unfavorable method for clinical applications. EVs have great potential for further optimization through, for instance, the addition of targeting sequences. 

In this work, we loaded EVs via the transient transfection of donor cells with pDNA/CPP complexes. pDNA/CPP complexes internalize cells, where a fraction of them is encapsulated to the EVs. First, we confirmed the proof of concept by measuring the labeled pDNA from EVs. Next, to evaluate the efficacy of our method, the loaded EVs were used to introduce genetic material into recipient cells, and luciferase reporter protein’s expression levels were measured from the delivered EV-encapsulated pDNA in recipient cells. We found that the EV-encapsulated pDNA resulted in efficient protein expression in the recipient cells up to ×10^4^ logs compared to the untreated cells. Based on in vivo experimental data, we observed approximately three times lower toxicity compared to the naked pDNA controls. Therefore, we conclude that the proposed method for using cell-penetrating peptides for loading plasmid DNA into secreted extracellular vesicles is efficient, and the use of pDNA-loaded EVs as native delivery vehicles reduces the toxic effects associated with traditional gene therapy methods.

## 2. Materials and Methods

### 2.1. Cell Culture Maintenance

Adherent CHO-K1 (Chinese Hamster Ovary cells), N2a (mouse neuroblastoma cells), HEK293 (human embryonic kidney cells), and A549 (human lung carcinoma epithelial cells) cell lines were cultured in a humidified environment at 37 °C, 5% CO_2_, and cultivated in Dulbecco’s Modified Eagle’s Medium (DMEM) (Sigma-Aldrich Products—Life Science, St. Louis, MI, USA) or RPMI-1640 media (Sigma-Aldrich Products—Life Science, St. Louis, MI, USA) supplemented with 100 U/mL penicillin, 100 mg/mL streptomycin, 0.1 mM non-essential amino acids, 1.0 mM sodium pyruvate, and 10% (final) fetal bovine serum. The suspension HEK293FT cell line was cultured as a suspension cell culture in a humidified incubator at 37 °C, 8% CO_2_, and cultivated in Xell HEK TF media supplemented with 100 U/mL penicillin, 100 mg/mL streptomycin, and 6 mM GlutaMAX™. Transfection and EV-production media were fetal bovine serum-free.

### 2.2. Peptide Synthesis and Sequences

Peptides were synthesized according to our previously published protocol [22]. Briefly, CPPs were synthesized on an automated peptide synthesizer Biotage Initiator+ Alstra (Biotage, Uppsala, Sweden) using the fluorenylmethyloxycarbonyl (Fmoc) solid-phase peptide synthesis strategy with Rink-amide ChemMatrix resin (0.41 mmol g^−1^ loading). The peptides were purified via reversed-phase high-performance liquid chromatography on a C4 column (Phenomenex Jupiter C4, 5 μm, 300 Å, 250 × 10 mm) using a gradient of acetonitrile/water containing 0.1% TFA. The concentration of the peptides was determined based on dilutions of accurately weighed substances and the absorption of tyrosine, where applicable. 

Sequences of CPPs used in this work:

NF55 Stearoyl-AGYLLGO*INLKALAALAKAIL-NH2;
CPP NF 71 Stearoyl-HHYHHGO*ILLKALKALAKAIL-NH2;
CPP PF14 Stearoyl- AGYLLGKLLOOLAAAALOOLL-NH2.
O*—synthesis continued from the side chain instead of the alpha-amino group.

### 2.3. Transfection

Complexes containing the peptides and pDNA were formed according to the protocol in our previously published work [22]. In the current work, a firefly luciferase-encoding plasmid (pMC.BESPX-GLucFLuc2) and CD63-EGFP-encoding plasmid (AddGene pEGFP C2 62964) were used.

Briefly, the pDNA and peptide complexes were mixed in MilliQ water at a charge ratio of 1:2 or 1:4 with the CPPs in excess and incubated at room temperature for up to 40 min before being added to the cells. The charge ratio (CR) and pDNA loading dosage vary throughout the current study depending on the desired conditions of the specific experimental setup. 

### 2.4. Donor Cell Culture Transfection for the Quantification of Cy5-Labeled pDNA in Exosomes

pDNA was labeled with a Mirus Bio™ Label IT™ Nucleic Acid Labeling Kit (Mirus Bio LDD, WI, USA), with Cy5 fluorophore following the manufacturer’s instructions.

Briefly, 40,000 CHO-K1 cells per well or 50,000 N2a cells per well were seeded on a 24-well plate in DMEM on the 1st day. On the 2nd day, the medium was changed to OptiMEM, and pDNA/CPP complexes were added and incubated for 4 h. In this experiment, we formed complexes with CPP PF14 at CR 1:4 and 0.5 µg of pDNA per well. Next, the cells were washed, and a fresh medium was added followed by a 4 h chase period. 

### 2.5. Donor Cell Culture Transfection for the Quantification of Radio-Labeled pDNA Detected in Isolated EVs

First, 1,000,000 cells per 75 cm^2^ flask in 10 mL of DMEM were seeded on the 1st day. On the 2nd day, the medium was changed to OptiMEM, and pDNA/CPP complexes were added and incubated for 4 h. The 2nd medium was changed to OptiMEM, and pDNA/CPP complexes were added and incubated for 12 h. In this experiment, we formed complexes with CPP PF14 at CR 1:4 and 200 µg of pDNA per flask. Next, cells were washed, and a fresh medium was added followed by a 48 h chase period.

### 2.6. Donor Cell Culture Transfection for Recipient Cell Transfection with Conditioned Media

Briefly, 40,000 CHO-K1 cells per well, 50,000 N2a cells per well, or 50,000 HEK293 cells per well were seeded on a 24-well plate in DMEM, and 100,000 HEK293FT cells per well were seeded on a 24-well plate in a Xell HEK TF medium on the 1st day. On the 2nd day, media were changed to OptiMEM (Sigma-Aldrich Products—Life Science, St. Louis, MO, USA) for CHO-K1, N2a, and HEK293. Experiments with HEK293 were performed with the Xell HEK TF (Xell AG, Bielefeld, Germany) medium. pDNA/CPP complexes were added to transfection media and co-incubated with cells for 4 h. In the optimization step, we formed complexes with CPP PF14, NF71, and NF55 at CR 1:1, 1:2, 1:3, and 1:4. pDNA dosage varied from 0.05 to 0.75 µg of pDNA per well. Next, cells were washed, and fresh media were added, followed by a 4–30 h chase period.

### 2.7. Donor Cell Culture Transfection for Recipient Cell Transfection with Isolated EVs

For this procedure, 6,000,000 HEK293FT cells per well were seeded on a 6-well plate, or 10,000,000 cells per 175 cm^2^ flask were seeded in Xell HEK TF media. Next, pDNA/CPP complexes were added and incubated for 4 h. We formed complexes with CPP NF71 at CR1:2 pDNA, and dosage varied from 0.05 to 0.75 µg of pDNA per well. Next, cells were washed, and fresh media were added, followed by a 4–30 h chase period. pDNA dosage varied from 3 to 6 µg of plasmid per well/flask.

### 2.8. EV Isolation

#### 2.8.1. EV Production

Briefly, 10,000,000 suspension HEK293FT cells were seeded to 20 mL of the Xell HEK TF medium on transfection day. The cells were transfected with pDNA/CPP complexes at CR 1:2 and incubated for 4 h. Cell culture media (conditioned media) were collected into separate labeled tubes distinguished by conditions. The media were centrifuged for 30 min at 2000× *g* at 4 °C to remove cells and debris. Centrifugation was performed with Sigma 2-16p centrifuge, rotor 12071.

#### 2.8.2. Isolation of EVs via Polymer Precipitation

The cell-free conditioned media from the previous procedure were transferred to a new tube, and a 0.5 mL sample volume of a total exosome isolation reagent (from cell culture media) (Invitrogen, Waltham, MA, USA) was added to the sample and mixed via pipetting. Samples were incubated overnight at 4 °C. Next, the samples were centrifuged for 1 h at 10,000× *g* at 4 °C (Optima XE-90 Ultracentrifuge, rotor SW28, centrifuge tubes 326823). The supernatant was discarded. The pellet containing the EVs was resuspended in 1xPBS. 

#### 2.8.3. Isolation of EVs with Tangential Flow Filtration

A commercially available tangential flow filtration-based device (TFF-MV) was used (HansaBioMed Life Sciences OÜ, Tallin, Estonia) for EV purification. The next steps were performed according to the manufacturer’s instructions. Briefly, samples were injected through the filter into TFF-MV. Next, filtration was performed by pushing syringes until there was no more permeate running. Extracellular vesicles were recovered by injecting 1xPBS into the filter and collecting retentate. 

#### 2.8.4. Isolation of EVs with Size Exclusion Chromatography Columns

The sample was purified with the size exclusion chromatography column (HansaBioMed Life Sciences OÜ, Tallin, Estonia). Starting with 20 mL of conditioned media, 0.5 mL fractions were collected and analyzed according to the manufacturer’s protocol.

#### 2.8.5. Storage Conditions of Purified EVs

Samples were stored at 4 °C for up to one week, at −20 °C or −70 °C for long-term storage.

### 2.9. Quantification of the Luciferase Reporter Expressed from the Transfected pDNA in Cell Lysate

The detection of luminescence from transfected cells and the normalization of relative luminescence units (RLUs) to total protein content was performed according to our previously published protocol [23]. Briefly, the luminescence signal was detected with a GloMax^®^ 96 microplate luminometer equipped with GloMax^®^ 1.9.2 software (Promega, Madison, WI, USA) from the cell lysate containing transfected cells in 0.1% Triton X100 in 1xPBS. RLU values were converted to RLU/mg through normalization to the total protein in the cell lysate. For total protein detection, a Pierce™ BCA Protein Assay Kit (Thermo Fischer Scientific, Waltham, MA, USA) was used. Data were analyzed with GraphPad Prism for Windows (version 10.0.3, GraphPad Software, San Diego, CA, USA).

### 2.10. Quantification of Purified Exosomes with ELISA

A double-sandwich ELISA assay was performed for the quantitative and qualitative analyses of exosomes using an ExoTEST ready-to-use test kit for overall exosome capture, and quantification from the cell culture supernatant (HansaBioMed Life Sciences OÜ; Tallin, Estonia, Product Code: HBM-TRTK-POC/TC) was carried out according to the manufacturer’s protocol. 

### 2.11. Quantification of Purified EVs with Nanoparticle Tracking Analysis (NTA)

Nanoparticle tracking analysis (NTA) was performed using a Zetaview analyzer in the scattered mode (embedded laser 40 mW at 488 nm). The instrument was previously calibrated using polystyrene nanospheres of 100 nm, according to the manufacturer’s protocol. Samples were diluted in 1xPBS to a final volume of 5 mL, with a dilution factor to obtain a particle-per-frame value of 50–200 (reference value 120–200). For each sample, three measurement cycles were performed, scanning 11 cell positions in each cycle and capturing 30 frames per position using the following settings: camera sensitivity: 85, shutter: 100; cell temperature: 25 °C.

### 2.12. Quantification of Radio-Labeled pDNA from Transfected Cells

First, 10,000,000 adherent CHO-K1 cells were seeded in 15 mL of DMEM on transfection day. The cells were transfected with pDNA/CPP complexes at CR1:2 and incubated for 4 h in an OptiMEM medium. Next, cells were washed 2 times, and a fresh medium was added, followed by a 48 h chase period. Cell culture media (conditioned media) were collected into separately labeled tubes distinguished by conditions. The media were centrifuged for 30 min at 2000× *g* at 4 °C to remove cells and debris. Centrifugation was performed with Sigma 2-16p centrifuge, rotor 12071.

Radio-labeled pDNA was produced with the Nick Translation System (Invitrogen, Walth, MA, USA) using [α32P]dCTP according to the manufacturer’s protocol. EV isolation was performed using the polymer precipitation method. Measurement was performed with a PerkinElmer Tri-Carb^®^ Liquid Scintillation Analyzer 2810 equipped with QuantaSmart™ software (version 2810TR, PerkinElmer, Turku, Finland).

### 2.13. Flow Cytometry

Cells were co-incubated with pDNA/CPP complexes for 4 h, followed by a chase period of 4 h. Exosomes were isolated with the total exosome isolation reagent (from cell culture media) (Invitrogen, Walth, MA, USA, cat.nr.4478359). The pDNA used in experiments with immuno-stained EVs was labeled with a Label IT^®^ Nucleic Acid Labeling Kit, Cy^®^5 (Mirus Bio LDD, WI, USA). Exosomes were labeled with a CD63 Monoclonal Antibody (MEM-259), Alexa Fluor™ 488 (Thermo Fischer Scientific, Waltham, MA, USA), according to the manufacturer’s protocol.

Flow cytometry was performed using an Attune™ NxT Flow Cytometer (Invitrogen, Walth, MA, USA) equipped with a 488 nm argon laser. The viable cell population was determined from a plot of forward-scattered vs. side-scattered light. The Attune™ NxT Software (version 3.2.1, Invitrogen, Walth, MA, USA) was used to analyze a minimum of 3000 events per sample. The area scaling factor was 1.28, with threshold values of SSC 0.3, FSC 220, SSC 360, BL1/GFP 460, RL1/APC/Cy5 520.

### 2.14. In Vivo Experiments

For in vivo transfection, purified pDNA-loaded exosomes or conditioned media were used. The luciferase reporter gene expression levels were evaluated after a single injection from the whole-tissue homogenate postmortem at a 24 h time point post-administration. The time point was chosen based on our observations from previous works. Animal experiments and procedures were approved by the Estonian Laboratory Animal Ethics Committee (approval no 203, 22 September 2021). For in vivo experiments, male and female 8-week-old BALB/c mice were used. 

### 2.15. Data Analysis

Data analysis was performed in GraphPad Prism 10.0.3.

## 3. Results

### 3.1. Proof-of-Concept Study of EV Loading via the Transient Transfection of Donor Cells with pDNA/CPP Complexes

**Figure 1:**
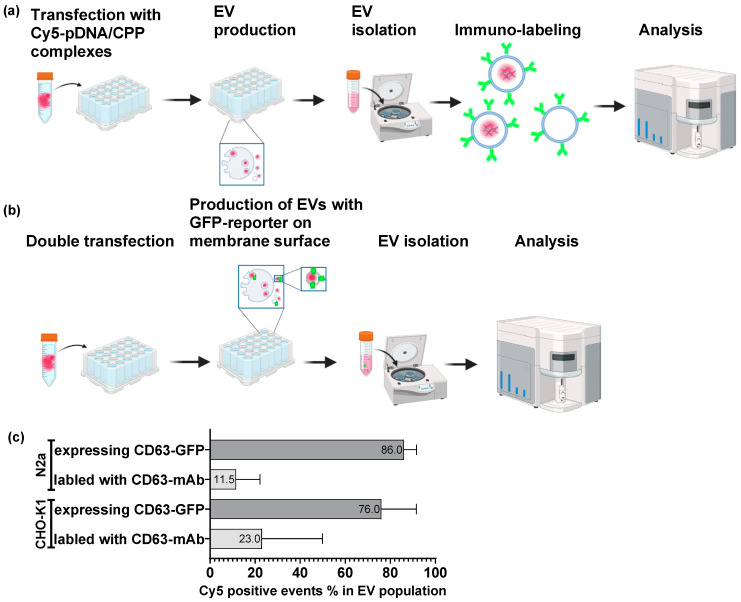
Quantification of Cy5-labeled pDNA in exosomes purified from donor cells’ conditioned media assessed via flow cytometry: (**a**) schematic representation of experimental setup of quantification of Cy5-labeled pDNA in total EV population (immuno-labeling protocol); (**b**) schematic representation of experimental setup of quantification of Cy5-labeled pDNA in total EV population (double transfection protocol); (**c**) flow cytometry assay. pDNA was labeled with Cy5 dye, and exosomes were either labeled with exosome-specific CD63 mAb or expressed CD63-GFP protein. pDNA containing EV population was gated if Cy5 and CD63 signal were present (Cy5+/GFP+). Transfection was performed with CPP PF14 in CHO-K1 or N2a cell lines. N = 2. Results are shown in % of the total EV population. Panel (**a**,**b**) were created with BioRender.com, acessed on 20 May 2023.

**Figure 2:**
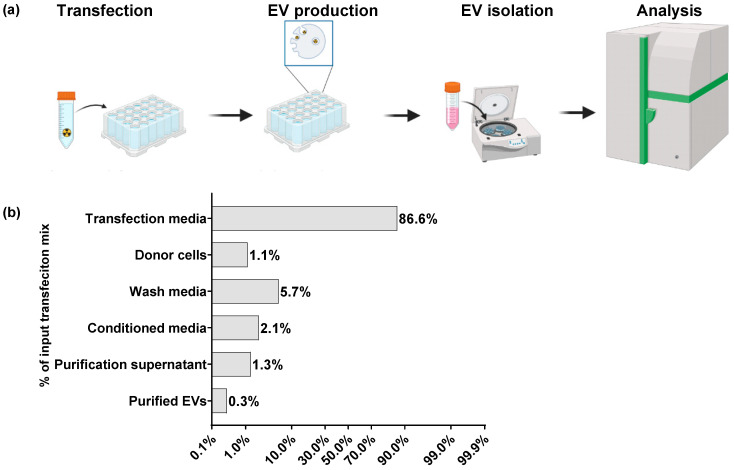
Quantification of radio-labeled pDNA in EVs purified from donor cells’ conditioned media: (**a**) schematic representation of experimental setup of quantification radio-labeled pDNA in total isolated EV population; (**b**) quantification of radio-labeled pDNA detected in EVs assessed by measuring [α32P]dCTP sample signals. CPP PF14 was used for complex formulation. The CHO K1 cell line was used as a donor cell line for loaded EV production. N = 3. Results are shown as % of the total sample input of labeled pDNA. Panel (**a**) was created with BioRender.com acessed on 20 May 2023.

For the proof-of-concept study of the proposed EV-loading method, donor cells were incubated with labeled pDNA/CPP complexes, followed by the substitution of transfection media with fresh media, and EV production. Thereafter, EVs were isolated from the conditioned media using the polymer precipitation method. To measure the proportion of labeled pDNA in the total exosome or total EV population, the samples were analyzed using either flow cytometry or radio-signal measurement (Figure 1a,b and Figure 2a). 

#### 3.1.1. Quantification of Cy5-Labeled pDNA in Exosomes

First, pDNA was covalently labeled with Cy5-dye before complex formation. This Cy5-labeled pDNA was complexed with CPP PepFect 14 (PF14) prior to transfection. These complexes were used for donor cells’ transient transfection via co-incubation. The loading efficacy of this method was assessed in CHO-K1 and N2a cells via flow cytometry.

The total exosome population was characterized using the following two approaches: 

1. Isolated EVs were immuno-labeled with an exosome-specific CD63 monoclonal antibody (CD63 mAb) (Figure 1a); 

2. Donor cells were co-transfected with two plasmids: model CD63-GFP plasmid and reporter Cy5-labeled plasmid. In case of efficient double transfection cells are producing exosomes with CD63-GFP exposed on exosome surface and are containing Cy5-labeled plasmids (Figure 1b).

In both CHO-K1 and N2a cell lines, we observed high loading efficacy for the labeled pDNA, ranging from 76% to 86%, when cells were co-transfected with CD63-GFP and Cy5-labeled plasmids. However, when EVs were characterized exosomes via CD63 immuno-labeling, the detected loading efficacy was significantly lower, at 23%, and for the N2a cell line, this was 11.5%. We hypothesized that the lower loading efficacy in the donor N2a cells compared to CHO-K1 cells may be caused by suboptimal conditions and/or associated with N2a cells being more resistant to transient transfection (Figure 1c). 

#### 3.1.2. Quantification of Radio-Labeled pDNA in Purified EVs

Next, the pDNA was labeled with [α32P]dCTP and used to form pDNA/CPP complexes (Figure 2a). This labeled pDNA was complexed with CPP PF14 before donor cells’ transfection. The efficacy of the loading method was assessed in CHO-K1 cells by measuring the radio signal. Our primary objective was to assess the effectiveness of transfection by measuring the amount of cargo radio-labeled pDNA in the total EV population. For this, throughout the process, we collected samples at various stages of EV production and purification to evaluate the recovery rate of nucleic acid (NA).

Approximately 87% of the initial input material signal was detected from the transfection media, and ~6% was detected in the media used for cell wash, indicating the efficacy of the donor cells’ overall transfection. Notably, 2.1% was detected in the unpurified conditioned media, and 1.1% was detected in donor cells at the harvesting step. The final purified EV fraction contained 0.3% of the initial cargo (Figure 2b). Thus, despite the need for optimization in the transfection process, our findings confirm that the method used for loading EVs via the transient transfection of donor cells is feasible.

### 3.2. Transfection of Recipient Cells with the Donor-Produced pDNA-Cargo-Loaded EVs Containing the Conditioned Media 

The general workflow in terms of the experimental setup remained constant throughout this series of experiments of this section. In brief, donor cells were transfected with pre-formed pDNA/CPP complexes, followed by transfection, cell wash, and the addition of fresh media. A chase period ensued during which donor cells produced cargo-containing EVs, releasing them into the media. Subsequently, we centrifuged the collected conditioned media and added them to recipient cells for incubation. Finally, we analyzed the reporter luciferase protein signal from both donor and recipient cell lysates.

#### 3.2.1. Development of Donor Cell Transfection Protocol for pDNA-Loaded EV Production

We evaluated the transfection efficacy in adherent CHO-K1 and HEK293 cell lines. pDNA/CPP complexes were formed with CPPs previously optimized in our laboratory for particular cell lines. Specifically, for the CHO-K1 cells, we used CPP PF14, and for HEK293 cells, complexes were formed with CPPs NickFect 55 (NF55) and NickFect 71 (NF71). 

Interestingly, the transfection of CHO-K1 cells yielded a significantly lower reporter signal, approximately three logs lower than the HEK293 cells (Figure S1). HEK293 cells, originally isolated from human embryonic cells, are of greater clinical interest compared to CHO-K1, which originates from Chinese hamsters. Given this consideration, we opted to further optimize our protocol using the HEK293 cells.

When the HEK293 cells were transfected with complexes using NF55 or NF71, there was no significant difference in the levels of luciferase reporter protein, as measured in relative light units per milligram (RLU/mg) (Figure S1). Notably, CPP NF71 has demonstrated efficiency as an NA transfection vehicle for both in vitro and in vivo applications. Furthermore, recent findings have shown that CPP NF71 has lower toxicity at earlier detection time points compared to other transfection methods. This promising characteristic makes NF71 an excellent starting point for the development of a method for producing pDNA-loaded EVs. Subsequent protocol optimization was carried out using CPP NF71.

#### 3.2.2. pDNA-Loaded EV Production in Suspension Cells

Next, we hypothesized that adopting suspension cell culture for production presents several advantageous features, such as the reduced consumption of consumables and an enhanced overall EV yield. To evaluate if and how the efficacy of donor cell transfection impacts the loaded EV production, we conducted parallel experiments, where adherent donor cells and suspension donor cells were transfected with the same amount of pDNA. Subsequently, the conditioned medium from each cell line was collected, centrifuged, and transferred to recipient cells. The luciferase reporter signal was measured from the donor and recipient cell lysates and normalized to the total protein. 

**Figure 3:**
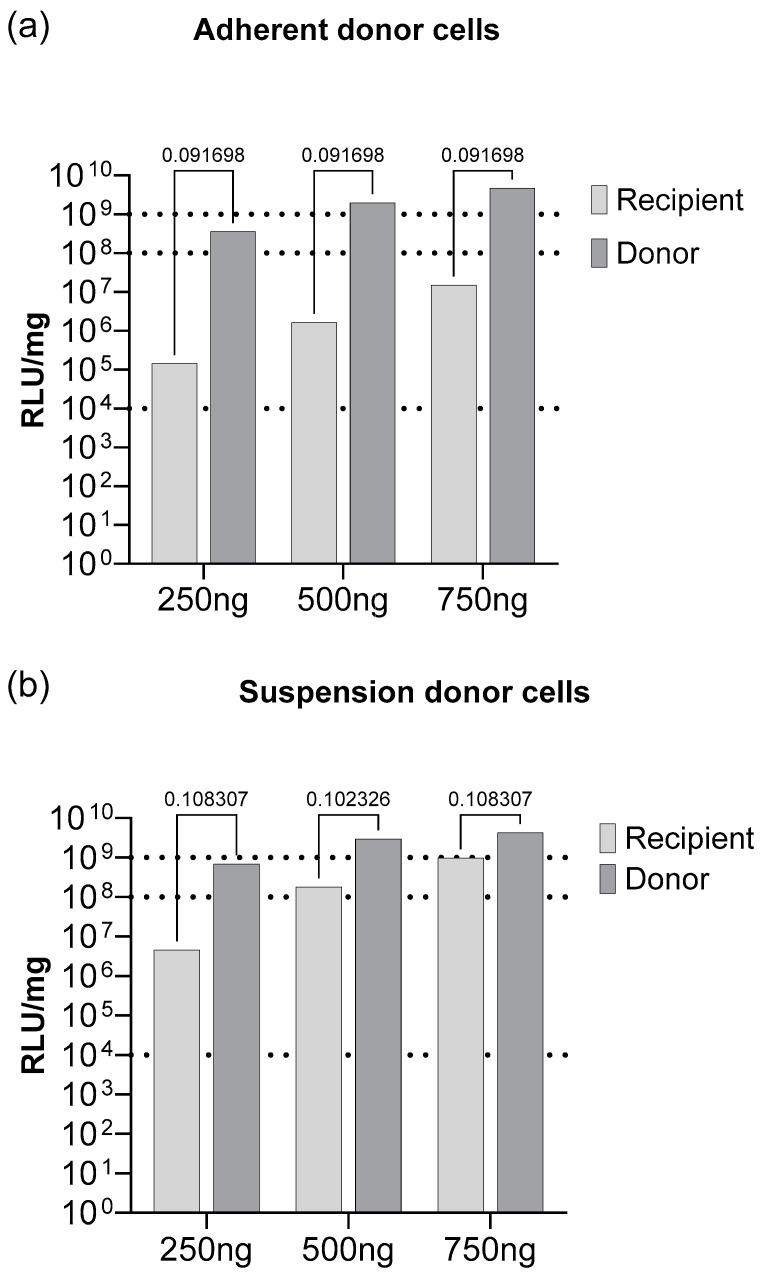
The signal level of luciferase reporter expressed in adherent recipient CHO-K1 cells after transfecting with pLuc-loaded EVs, extracted from either (**a**) an adherent donor CHO-K1 cell line, (**b**) or a suspension HEK293FT donor cell line. On the *x*-axis, the dosageof pDNA in pDNA/CPP complex per well used for transection is shown. N = 6. For statistical analyses, multiple unpaired *t*-tests were performed. Results are shown in RLU/mg and normalized to total protein in the lysate.

We observed high levels of reporter signal in both suspension and adherent donor cells, and the optimal result was observed in the suspension cells up to 1 × 10^10^ RLU/mg. When recipient cells were incubated with the conditioned media from the suspension donor cells, the reporter expression was only one log lower than the signal measured in donor cells (Figure 3a). The incubation of recipient cells with the conditioned media collected from suspension cells led to approximately two logs higher signal when compared to incubation with the conditioned media collected from adherent cells (Figure 3b). 

### 3.3. Scaling up EV Production in the Suspension Cell Line

The optimized dose of pDNA for small-scale production may not always be directly applicable to large-scale production [22]. Therefore, we optimized conditions for scaling up EV production in the suspension cells. Our results yielded no significant differences when evaluating various dosages, namely 3 mg, 4.5 mg, or 6 mg per 6,000,000 cells (Figure S2). 

In the subsequent phase of optimization, we explored variations in the transfection duration, chase periods, and pDNA dosage to enhance the loaded EV production (Figure S3). Our findings reveal that, in our system, a 4 h donor cell transfection period and a subsequent 4 h recipient cell incubation with conditioned media represent the optimal conditions.

### 3.4. Isolation and Characterization of Purified Extracellular Vesicles

Our next objective was to assess EV purification methods that are compatible with standard laboratory settings, avoiding the need for sophisticated equipment or intricate protocols. We collected conditioned media from donor cells and subjected them to purification using three distinct techniques, namely (1) polymer precipitation, (2) tangential flow filtration, and (3) size exclusion chromatography, following the respective manufacturer’s protocols.

**Figure 4:**
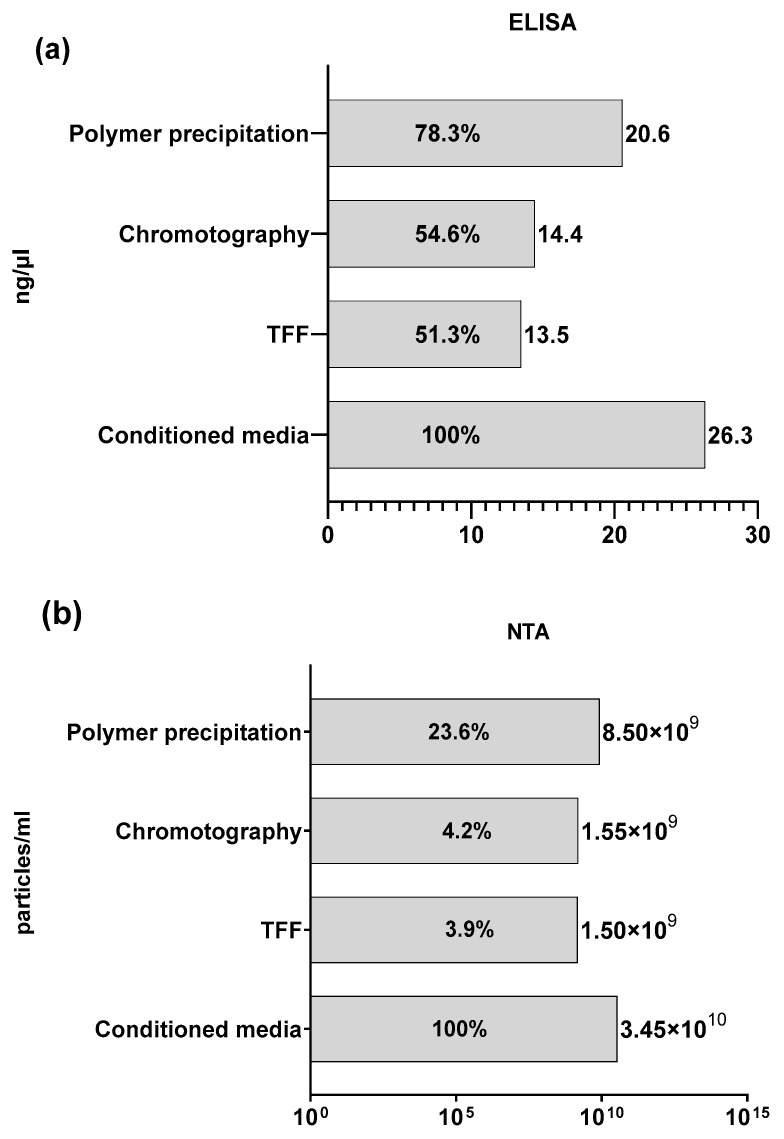
Comparison of EV isolation methods. Conditioned media were purified via polymer precipitation, tangential flow filtration, or size exclusion chromatography. Inside the bar % of sample measurements from conditioned media input are shown: (**a**) double-sandwich ELISA assay for quantitative and qualitative analyses of exosomes. Results are shown in ng/µL; (**b**) NTA for the determination of a size distribution profile of small particles with a specific diameter in liquid suspension. N = 1. Results are shown in particle/mL.

We utilized a double-sandwich ELISA assay to confirm the purified EVs as exosomes and to measure particle concentration in ng/µL. Additionally, nanoparticle tracking analysis (NTA) was employed to evaluate both the size distribution and particle concentration in particles/mL. NTA revealed an approximate 4–24% recovery rate of EVs after purification (Figure 4a), whereas ELISA indicated a recovery rate of approximately 51–78% for exosomes (Figure 4b). Notably, both assays demonstrated a higher recovery percentage when conditioned media were purified using polymer precipitation. It is essential to recognize that NTA primarily characterizes the particle count and size distribution of EVs, whereas ELISA provides insights into the exosome concentration within the sample. Our hypothesis is that the assessed purification methods are indeed optimized for exosome purification, effectively eliminating other types of microvesicles. We found that EV purification via polymer precipitation is the most efficient method in our system. Therefore, we selected this method for subsequent experimental setups.

### 3.5. Transfection of Recipient Cells with pDNA-Loaded Exosomes

Our next objective was to confirm if the purified exosomes can effectively transfect recipient cells. The loaded exosomes were generated following a protocol that had been previously optimized in this work. This process involved the transfection of donor cells with pre-formed pDNA/CPP complexes, followed by media conditioning. Subsequently, the exosomes were isolated from the conditioned media using polymer precipitation, characterized by ELISA, NTA, and the quantification of the total protein content.

In this series of experiments, in addition to HEK293FT cells, we introduced the A549 cells as a recipient in our experimental setup to assess the efficacy of the loaded EV transfection under more complex conditions. A549 cells are recognized as a challenging cell line to transfect. Specifically, we utilized 8.5 × 10^6^ purified exosome particles for transfection into 100,000 recipient HEK293FT cells or 50,000 recipient A549 cells, with 85 or 170 exosomes per cell, respectively.

**Figure 5:**
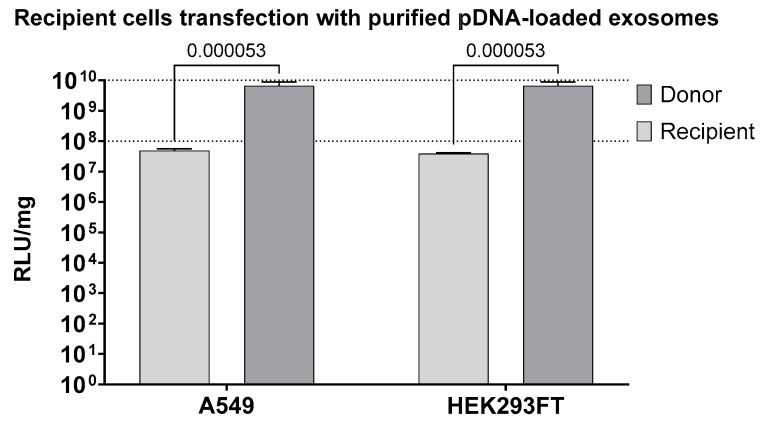
Cell transfection with isolated exosomes in the luciferase reporter assay. A549 and HEK293FT cell lines were used as recipient cell lines. The signal from the luciferase reporter was measured in recipient and donor cells. N = 6. For statistical analyses, multiple unpaired *t*-test was performed. Results are shown in RLU/mg, normalized to total protein in the lysate.

Our findings revealed a reporter protein signal in recipient HEK293FT cells, reaching high levels of up to 3.9 × 10^7^ RLU/mg. Similarly, in recipient A549 cells, we observed a robust reporter protein signal, reaching levels of up to 4.8 × 10^7^ RLU/mg (Figure 5). These data demonstrate that loaded exosomes can efficiently deliver cargo-pDNA to recipient cells, facilitating the expression of the reporter protein.

### 3.6. In Vivo Transfection with pDNA-Loaded EVs

Next, we were interested in the level of the reporter protein expression in recipient cells in an in vivo setting. The pDNA-loaded EVs were produced by applying two protocols, using either CPP PF14 and CHO-K1 cells or CPP NF71 and HEK293FT cells. The purified pDNA-loaded EVs were administered intravenously into 8-week-old female BALB/c mice via the tail vein.

**Figure 6:**
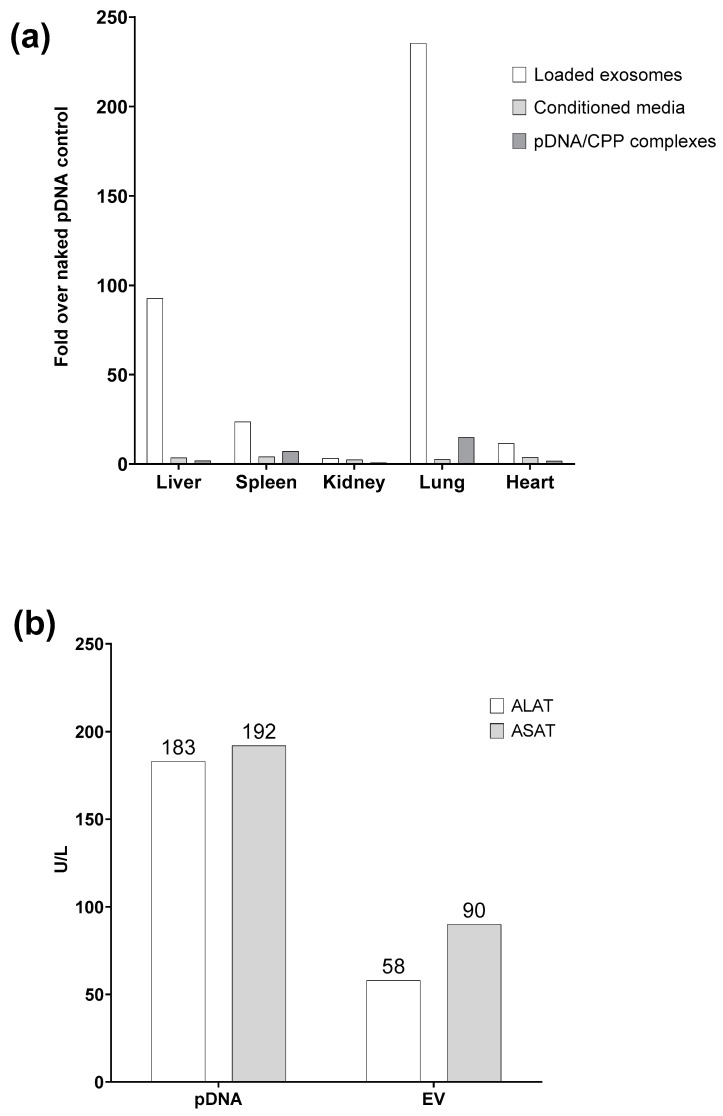
In vivo transfection. Eight-week-old female BALB/c mice were administered intravenously via the tail vein: (**a**) results of postmortem ex vivo tissue analysis are shown as the fold over pDNA control. Tissues were harvested 24 h after administration; (**b**) levels of ASAT and ALAT serum marker enzymes in whole blood were measured 24 h after administration. N = 1.

The ex vivo tissue analysis conducted postmortem revealed the successful expression of EV-encapsulated pDNA in mammalian tissues following intravenous administration. This was exclusively observed when CPP PF14 was used for loading EVs with pDNA (Figure 6a). Toxicity was evaluated via the analysis of serum marker enzymes ASAT and ALAT. The results revealed approximately three times lower levels of toxicity markers after EV administration compared to the lavels after naked pDNA control administration (Figure 6b).

## 4. Discussion

As a starting point for our proof-of-concept study, we conducted a series of experiments where the encapsulated labeled pDNA was measured from excreted EVs. Working on the lab-scale yield of purified EVs is low, and additional manipulations, such as DNase or RNase treatment, can result in sample loss. The assay results may be altered by a minimal amount of pDNA attached to the surface of EVs. However, the low levels of toxicity (see Figure 6b) suggest the minimal presence of free pDNA in the administered sample, if any.

First, pDNA was covalently labeled with Cy5 dye before complex formation with CPP PepFect 14 (PF14). These complexes were used for donor cells’ transient transfection. We applied two different methods for the characterization of the EV population, and the data revealed variations in the results. We speculate that the difference in the loading efficacy, as measured with the application of the two different characterization approaches, may be biased due to the differences in assay protocols. Immuno-labeling quantifies the percentage of cargo-containing exosomes within the total EV population. In the case of the double-transfection method, we characterized the EV population where the reporter protein was expressed and exposed on the EV surface, not counting the EVs produced by cells in the culture that do not express the reporter protein. We speculate that EVs produced by double-transfected donor cells are more likely to contain Cy5-labeled plasmids than the entire EV population. 

Next, we hypothesized that the labeled pDNA complexation with CPPs and incorporation into exosome can lead to a reduction in fluorescence intensity, which may alter the detection efficacy of the reporter Cy5. To address this issue, the pDNA was labeled with [α32P]dCTP (Figure 2a). We observed a significant difference between fluorescent- and radio-labeled pDNA signal measurements in the total exosome or EV population. Specifically, in the final isolated EV population, the positive signal for radio-labeled pDNA accounted for only 0.3% of the total input material, in contrast to the recovery rates of 11–86% observed for fluorescent-labeled pDNA (Figure 1c and Figure 2b). Our approach to measuring the proportion of radio-labeled pDNA involved tracking the pDNA recovery at each step, from donor cell transfection to EV purification (Figure 2b). On the other hand, when evaluating fluorescent-labeled pDNA, our focus was solely on assessing the success of cargo loading into EVs, without considering donor cell transfection efficacy (Figure 1c). Through the evaluation of radio-labeled pDNA, we were able to estimate the percentage of input material loaded into isolated EVs. However, it is important to emphasize that this method does not provide data on the percentage of cargo-loaded particles within the total EV population. Taking into consideration the labeled pDNA quantification in exosomes and EVs, we confirmed that these vesicles are capable of accommodating pDNA/CPP complexes. 

It is worth noting that the detection of labeled cargo is often used to evaluate transfection efficacy, and it is an initial indicator of the potential pDNA/CPP loading into the excreted EVs. However, it does not necessarily reflect the bioavailability and bioactivity of the cargo molecules. Even if the cargo is efficiently loaded into the transport vehicle, it may not impact the gene expression of recipient cells or have therapeutic effects. Therefore, we utilized pDNA expressing a reporter luciferase protein to assess the total reporter levels in recipient cells when pDNA-loaded EVs from donor cells’ conditioned media were employed [22,24,25]. 

Proceeding with method development, we speculate that maximizing the number of successfully transfected cells allows for optimal cargo-loaded EV production [22]. During the experimental setup development, we took into consideration the dependability of transfection efficacy on the used cell line, transfection conditions, EV production conditions, pDNA dose, and CPP features. These aspects affect the proportion of the loaded EVs and the general vesicle production. We performed a series of experiments for broader condition screening and protocol optimization, as well as to evaluate whether the conditioned media collected from donor cells can be used for recipient cell transfection. Next, we hypothesized that adopting a suspension cell culture for production presents several advantageous features, such as the reduced consumption of consumables and an enhanced overall EV yield. Our findings strongly support the notion that producing loaded EVs in suspension cells is more efficient, and thus we proceeded with the production of loaded EVs using the suspension HEK293FT cell line (Figure 3a,b). The optimization of pDNA dosage for small-scale production may not always directly apply to large-scale production [22]. Therefore, we optimized the conditions for scaling up EV production in the suspension cells. These optimized conditions are envisioned to be translated into industrial-scale production through careful dosage and transfection time optimization. These optimized parameters give us the foundation for scaling up EV production in our suspension cell line, thus facilitating its translation into industrial-scale production.

Next, we evaluated EV purification methods that are compatible with standard laboratory settings, avoiding the need for sophisticated equipment or intricate protocols. We compared three techniques: (1) polymer precipitation, (2) tangential flow filtration, and (3) size exclusion chromatography. The results were assessed using ELISA and NTA (Figure 4). We determined that EV purification via polymer precipitation is the most efficient method within our system. Therefore, we selected this method for subsequent experimental setups.

Our next objective was to confirm if the purified exosomes can effectively transfect recipient cells, leading to the expression of the reporter plasmid at reasonable levels. The loaded exosomes were generated following a protocol that had been optimized for scaled-up production. This process involved transfecting donor cells with pre-formed pDNA/CPP complexes, followed by media conditioning, exosome isolation, characterization using ELISA, nanoparticle tracking analysis (NTA), and the quantification of the total protein content.

Our results demonstrate that loaded exosomes can efficiently deliver cargo-containing pDNA to recipient cells, facilitating the expression of the reporter protein. We observed no significant difference between HEK293FT and A549 cell lines, both resulting in a reporter protein level of ~4 × 10^7^ RLU/mg (Figure 5).

Next, we were interested in the level of the reporter protein expression in recipient cells in an in vivo setting. The reporter protein expression was analyzed via ex vivo tissue analysis, which was conducted postmortem. Compared to the mice lung tissue administrated with the naked pDNA control, the signal measured from the lungs of mice administrated with pDNA-loaded EVs was ~250 times higher. Notably, the administration of purified EVs diluted in PBS resulted in a lower toxic effect on mice than administration with pDNA controls. Lower toxicity was evident from the analysis of serum marker enzymes ASAT and ALAT, which indicated toxicity levels approximately four times lower than those observed with the naked pDNA (Figure 6b). It is important to highlight that the mice displayed no noticeable adverse reactions in response to the administration of EVs. In conclusion, our findings demonstrate that intravenously administered EV-encapsulated pDNA can successfully lead to a reporter protein expression in mammalian tissues. Moreover, the use of CPP PF14 for EV loading with pDNA appears to be particularly effective in achieving this outcome while minimizing toxicity, as evidenced by the serum marker enzyme analysis and the absence of visible adverse reactions in mice.

## 5. Conclusions

In this work, we developed and validated a method that can be developed for a non-toxic drug delivery system. We confirmed the efficacy of the production of pDNA-cargo-loaded exosomes in mammalian cells via donor cell transfection with pre-formed pDNA/CPP complexes. We proposed a potent approach with several advantages. First, our exploration data of transfection with pDNA-loaded EVs and investigation of utilization of the targeting sequences on EV surface can be expanded for methodology advancement. Such development holds the potential to further improve the efficacy of drug delivery systems. Furthermore, this method leverages inherent features of low toxicity and high biocompatibility, making it a promising candidate for clinical applications. In in vivo experiments, we observed minimal toxicity, paving the way for safe drug delivery. In conclusion, our work not only presents a robust method for non-toxic drug delivery but also highlights its potential for translation into clinical practice. With continued optimization and refinement, this approach offers exciting prospects for the development of safer and more effective therapeutic interventions, including other types of cargo, such as mRNA or small proteins.

## Data Availability

All data generated or analyzed during this study are included in this published article (and its supplementary information files). Raw data can be made available from the corresponding author upon reasonable request under MTA.

## Supplementary Materials

The following supporting information can be downloaded at: https://www.mdpi.com/article/10.3390/biom13121751/s1, Figure S1: Cell line and CPP evaluation for EV production protocol optimization; Figure S2: pDNA dosage optimization for scale-up protocol optimization; Figure S3: Transfection, chase period dosage optimization for scale-up protocol development.
