# Supplementary material for: A Method for Using Cell-Penetrating Peptides for Loading Plasmid DNA into Secreted Extracellular Vesicles
Source: Biomolecules. 2023 Dec 6;13(12):1751. doi: 10.3390/biom13121751 (PMC10741998; doi:10.3390/biom13121751)
Supplement: Supplementary file 1 [file biomolecules-13-01751-s001.zip › biomolecules-2730759-supplementary.pdf]

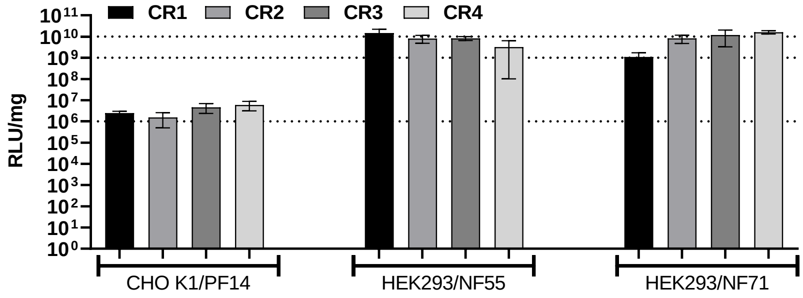

Figure S1. Cell line and CPP evaluation for EV production protocol optimization. Measurement of the luciferase reporter signal in transiently transfected donor cells. For the CHO-K1 cells we used CPP PF14, and for HEK293 cells NF55 or NF71. On x-axes cell line and respective CPP and pDNA/CPP charge ratio used for transfection are shown. On y-axes results in RLU/mg are shown. Results are normalized to total protein in the lysate. Experiments were performed in 96-wp format, with 100 ng pDNA dosage per well, and in serum-free medium.

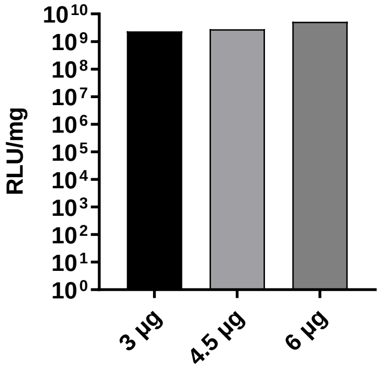

Figure S2. pDNA dosage optimization for scale-up protocol optimization. Measurement of the luciferase reporter signal in transiently transfected donor cells. On x-axes dosage of pDNA per well is shown. On y-axes results in RLU/mg are shown. Results are normalized to total protein in the lysate. Experiments were performed in 175 cm^2 bottles.

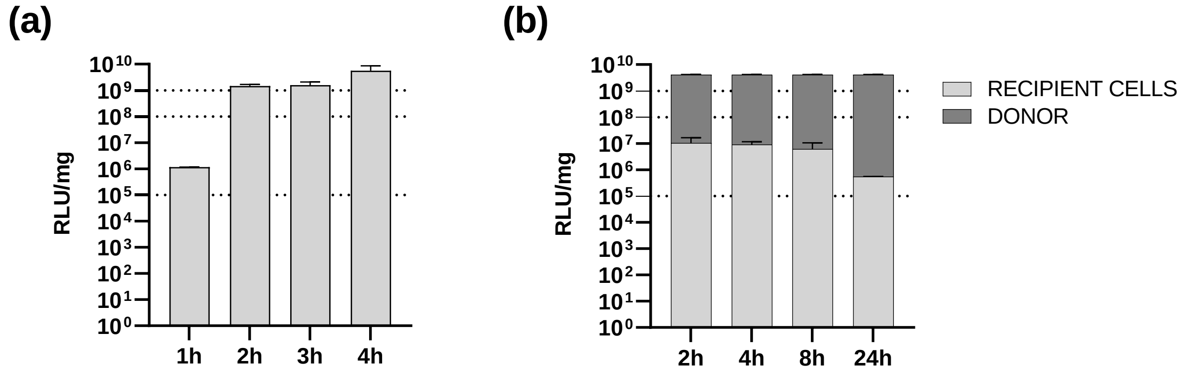

Figure S3. Transfection, chase period dosage optimization for scale-up protocol development. Measurement of the luciferase reporter signal in transiently transfected donor cells. (a) Transfection period of donor cells. (b) Chase period of EV production, with following recipient cells incubation with conditioned media. On y-axes result in RLU/mg are shown. Results are normalized to total protein in the lysate. Experiments were performed on 6 cm^2 plates.
